# Exploring the use of digital media to support meaningful activities for people living with dementia: A qualitative study

**DOI:** 10.1177/14713012251330689

**Published:** 2025-03-29

**Authors:** Mariana Ramalhete, Rita Maldonado Branco, Soraia Teles, Oksana Tymoshchuk, Rita Oliveira, Joana Quental, Oscar Ribeiro

**Affiliations:** Department of Education and Psychology, 56062 University of Aveiro, Portugal; Research Institute for Design, Media and Culture (ID+), 56062 University of Aveiro, Portugal; Department of Communication and Art, 56062 University of Aveiro, Portugal; Center for Health Technology and Services Research (CINTESIS@RISE), Portugal; Department of Behavioral Sciences, School of Medicine and Biomedical Sciences, University of Porto (ICBAS-UP), Portugal; Digital Media and Interaction Research Centre (DigiMedia), Portugal; Department of Communication and Art, 56062 University of Aveiro, Portugal; Digital Media and Interaction Research Centre (DigiMedia), Portugal; Department of Communication and Art, University of Aveiro, Portugal; Research Institute for Design, Media and Culture (ID+), 56062 University of Aveiro, Portugal; Department of Communication and Art, 56062 University of Aveiro, Portugal; Center for Health Technology and Services Research (CINTESIS@RISE), Portugal; Department of Education and Psychology, University of Aveiro, Portugal

**Keywords:** dementia, meaningful activities, digital media, internet use, focus groups, interviews

## Abstract

Dementia is a progressive and neurodegenerative condition that leads to a gradual deterioration of the individual’s functional capacity and social relations. Engaging in meaningful activities is considered an effective approach to maintaining and increasing the well-being of people living with dementia. Digital media has the potential to improve the quality of life for people living with dementia, allowing them to engage in activities that are personally meaningful. This study sought to understand the needs and preferences people with early-stage dementia living in Portugal have for receiving information on meaningful activities. It also explored their relationship with technologies and digital media. Focus groups and interviews were carried out with people living with dementia (*n* = 21), informal carers (*n* = 9) and healthcare professionals (*n* = 8). Descriptive statistics were used for sample characterization and the *verbatim* transcriptions of interviews and focus groups were subjected to inductive thematic analysis. We developed three main themes: (i) engagement of people living with dementia in meaningful activities; (ii) experiences of people living with dementia with technology and digital resources; and (iii) the importance of a support network. This last theme is associated with the first two. The study identified several meaningful activities, such as household chores and intellectual hobbies, some of which were mediated by technology. People living with dementia reported to primarily use digital media, particularly computers and smartphones, for socialization and entertainment. The barriers identified for technology use and engagement in meaningful activities were both related to mobility problems and cognitive complaints. The support network emerged as essential for the use of digital technologies and engagement in meaningful activities. This study highlights a need to further research and design digital media that offer the opportunity for people living with dementia to be informed and engaged in meaningful activities.

## Introduction

Dementia is one of the major causes of dependency among older people worldwide (WHO, 2021). It has been estimated that, in 2019, approximately 14.1 million people in Europe were living with dementia (WHO, 2021) with Portugal ranking fourth among European countries with 21 cases per 1000 inhabitants (OECD, 2021). The prevalence of dementia in Portugal is expected to increase and reach about 5% of the population in 2080, particularly affecting older women (Alves et al., 2024).

Individuals in pre-dementia stages might present with mild cognitive impairment, considered a prodromal stage of dementia (Gauthier et al., 2006); or alternatively, subjective cognitive decline despite healthy limits in cognitive tests (Scheltens et al., 2016). When comparing these three clinical groups, Mank et al. (2022) reported that people along the continuum of Alzheimer’s disease present less life satisfaction than people with mild cognitive impairment and subjective cognitive decline, with reduced participation in meaningful activities as a major explaining factor.

The term ‘meaningful activity’ is commonly used in dementia literature, however there is a lack of consensus on its definition and measurement. A recent concepts analysis of the term *meaningful activity for older adults with dementia* (Tierney & Beattie, 2020) identified five defining attributes: enjoyable, suited to the individual, related to personally relevant goals, engaging, and able to express and reinforce one’s identity. Although engaging in meaningful activities is considered an effective nonpharmacological approach to maintain and increase well-being in people living with dementia (Regier et al., 2017), there is a lack of knowledge regarding which activities are helpful and how to best identify and facilitate them.

Digital and assistive technologies are promising ways to support their engagement in meaningful activities, improving well-being, quality of life and relationships among people living with dementia (Bradley et al., 2023; Goodall et al., 2020). Goodall et al. (2020) categorized assistive technologies used to promote individualized meaningful activities into four main purposes: reminiscence/memory support, behavior management, stimulating engagement, and communication support. Previous studies show that people living with dementia can express their preferences consistently and can learn how to use new digital technologies (Bradley et al., 2023; Span et al., 2013). However, there has been a lack of research on the development of technologies that promote and support leisure activities (Evans et al., 2015; Joddrell & Astell, 2016; Meiland et al., 2017), an important need area in dementia care. In addition, not all studies actively involve people living with dementia as stressed by the World Health Organization: “(…) 43% of countries report that people living with dementia are not at all involved in the research development process” (WHO, 2021, p. 230).

In fact, the lack of meaningful involvement of people living with dementia, informal carers, and healthcare professionals in the design of technology seems to be a reoccurring limitation (Bradley et al., 2023; Meiland et al., 2017; Span et al., 2013; Topo, 2008). An important element of involving people living with dementia in technology design is understanding their needs and preferences. This can be achieved through initial exploratory meetings (e.g., focus group format) (Wilson et al., 2022). Due to the role informal carers and healthcare professionals play in supporting people living with dementia, involving them in the focus groups phase ensures data richness and helps to achieve a generalized view of the themes covered.

This paper presents a study that aims to understand the needs and preferences of people with early-stage dementia living in Portugal regarding how to receive information on meaningful activities and their relationship with websites and apps pertaining to meaningful activities. This study was conducted under the scope of the project “Design for a humanized communication of dementia: co-creation of an information platform about engaging in meaningful activities” (DECOHDE), which aims to study and explore the design of information resources that are suitable, accessible, attractive and comforting for people living with dementia in Portugal, particularly about meaningful activities (Branco et al., 2022, 2024).

## Method

### Study design

In this qualitative study, separate focus groups and interviews were conducted with healthcare professionals, informal carers, and people living with dementia. Semi-structured interviews were carried out only when it was not possible to form a group of participants due to their schedule restrictions. The COREQ guideline (Tong et al., 2007) for reporting qualitative results was used (see Supplemental File).

### Participants and recruitment

A total of 38 people participated in this study. The recruitment of people living with dementia and informal caregivers was conducted using convenience sampling via the national dementia association (Associação Alzheimer Portugal) and local dementia support networks. These organizations were responsible for referring participants to the study upon their consent. Referred individuals were then contacted by the first author via phone call or email, and information about the study was sent along with a request for consent to participate (online form). Healthcare professionals were recruited by convenience considering their work experience with people living with dementia and invited via e-mail with information about the study. Invitations were sent to professionals in the researchers’ contact network who collaborated on previous studies and community intervention projects in the field of dementia. These included medical doctors, nurses, neurologists, psychologists, neuropsychologists, psychiatrists, social workers and gerontologists. The aim was to recruit participants across the country, though respondents were mostly from northern Portugal. Table 1 shows details of inclusion criteria for participants.

**Table 1.** Inclusion criteria for participants.

Participant	Inclusion criteria
People living with dementia	Formal diagnosis of dementia, or mild cognitive impairment.
	Be able to use the Internet independently or with support/supervision.
	Be able to read and understand information in Portuguese.
Informal caregiver	Minimum age: 18 years old.
	Have been providing unpaid care for a person with dementia for at least 6 months.
	Be able to use the Internet independently.
Healthcare professional	Background in psychology, gerontology, occupational therapy, social work, education (social), medicine or nursing.
	Have provided psychosocial support to people living with dementia, particularly regarding meaningful activities.

### Materials and data collection

Focus groups sessions and semi-structured interviews were carried out between December 2023 and May 2024. Three focus groups and five individual interviews were conducted with people living with dementia (*n* = 21); one paired and seven individual interviews with informal caregivers (*n* = 9); and two focus groups and one individual interview with healthcare professionals (*n* = 8). Characteristics of all the participants are displayed in Table 2. Focus groups and interviews with people living with dementia took place at the dementia support facilities they attended, at agreed schedules. One interview with one person with dementia was conducted online, via video conference call. Due to participants’ schedules, focus groups and interviews with healthcare professionals and informal carers were all conducted via video conference call.

**Table 2.** Characterization of the participants at the time of inclusion.

Healthcare Professionals (*n* = 8)	
Sex, male/female	0/8
Age, (median)	47.5
Years of professional experience (median)	17.5
Position (*n*)	
Gerontologist	2
Occupational Therapist	2
Psychologist	4
People living with Dementia (*n* = 21)	
Sex, male/female	5/16
Age (median)	87.5
Diagnosis (*n*)	
Alzheimer	3
Vascular dementia	3
Unspecified	15
Time since diagnosis, years (*n*)	
<1	11
1–2	4
2–3	2
>5	2
Unknown	2
Internet use, frequency (*n*)	
Every day	4
At least once a week	4
Less than once a week	2
Never	11
Internet use, device (*n*)	
Computer	7
Smartphone	3
Informal Caregivers (*n* = 9)	
Sex, male/female	4/5
Age (median)	56.5
Relation to people living with dementia (*n*)	
Wife/husband/partner	2
Son/Daughter	6
Daughter-in-law/Son-in-law	1
Caregiving experience, years (*n*)	
<1	4
2–3	2
3–4	1
>5	2

The focus groups and interviews followed semi-structured guides previously developed by the research team for this study (see Supplemental File). The guides covered people living with dementia and informal carers experience and behavior while using digital media, as well as their preferences regarding means to receive information about meaningful activities and its type of language and format (e.g., articles, Q&A, images, videos). The focus group and interviews were conducted in Portuguese by the first author, a junior researcher with a master’s degree in health psychology and neuropsychology, with the support of a senior researcher specialized in clinical psychogerontology (OR) and a senior researcher specialized in design for people living with dementia (RMB). Focus groups ranged from three to six participants, and each focus group met for one session. The session started with a presentation of the study, and the participants were encouraged to interact with each other. Field notes were taken to guide subsequent analysis.

Each focus group session lasted between 40 to 60 min, and interviews lasted around 20 min each, to minimize fatigue. Focus groups with healthcare professionals lasted approximately 90 min each. The semi-structured interviews held with one healthcare professional and nine informal caregivers lasted between 20 and 40 min.

Interview and focus group data were digitally recorded and transcribed *verbatim*. The data recordings were deleted after the transcription and text data was anonymized.

### Ethics

This study received approval from the Research Ethics Committee of the University of Aveiro, Portugal (Ref. 06-CED/2023) on March 29, 2023. As mentioned previously, people living with dementia were recruited through the national dementia association and local dementia support networks, who were responsible for confirming the participants’ diagnosis and capacity to give informed consent. All participants needed to be able to give informed consent to take part in the study. Although the study information and consent forms were sent and filled out prior to the research activities, they were also reinforced verbally to the participants in the beginning of the focus groups or interviews. It was outlined that participation was voluntary and that participants could withdraw from the session at any time.

### Data analysis

An inductive thematic analysis was undertaken using WebQDA (Sousa et al., 2019), a software program for qualitative analysis. The analysis followed the six-step approach outlined by Braun and Clarke (2006). Focus groups and interviews with people living with dementia, informal caregivers and healthcare professionals were analyzed together to provide a comprehensive view of the content of the entire data set. This method is beneficial in exploratory research, and when investigating an under-researched area (Braun & Clarke, 2006, 2022). The first author coded the focus group and interview data. The coding and interpretation of the findings was discussed by the research team (co-authors) who speak both Portuguese and English.

## Findings

Following an inductive thematic analysis, we developed three themes based on the interviews and focus groups: (1) engagement of people living with dementia in meaningful activities; (2) experiences of people living with dementia with technologies and digital resources; and (3) the importance of a support network. This section structure follows the main themes and sub-themes of the analysis, described in Table 3. To illustrate the findings and provide an in-depth understanding of the results, participants’ quotes included in this paper were translated posterior to the analysis by the first author from Portuguese to English. The accuracy of the translation was checked by the research team.

**Table 3.** Themes and sub-themes that emerged from the analysis.

Themes	Subthemes
Engagement of people with dementia in meaningful activities	Identifying meaningful activities (past and current)
	Barriers to maintaining meaningful activities
Experiences of people with dementia with technology and digital resources	Relationship with digital technology
	Accessing information online
	Preferences on information format
The importance of a support network	

The first theme details participants’ perceptions about the importance of people living with dementia engaging in meaningful activities. A wide range of meaningful activities, including some mediated by technology (i.e., writing in forums) were identified. The most reported activities included daily domestic chores (i.e., cooking, household chores) and intellectual activities (i.e., reading, puzzles, games). Intellectual activities were both accessed physically and digitally. While games were accessed more digitally, books and puzzles were accessed physically. Physical (i.e., lack of mobility) and cognitive (i.e., memory complaints) barriers were both associated with low engagement in meaningful activities and technology use. Healthcare professionals identified a lack of appropriate resources as an environmental barrier.

The second theme details participants’ perceptions about the experiences of people living with dementia with digital media and Internet use. Most participants with dementia were not familiarized with digital media and Internet use; however, some participants used computers for socializing and searching for news or information related to leisure activities. They didn’t express a preference for a specific format for information on meaningful activities. Video was considered as being easier for people living with dementia to access and understand by informal carers and healthcare professionals.

The third theme is associated to the two themes presented above and refers to the participants’ perceptions about the importance of a support network. Participants with dementia reported that they often rely upon their informal and formal carers to help with digital media use and to facilitate engagement in meaningful activities. There is an emphasized concern by informal carers and healthcare professionals in identifying and providing suitable meaningful activities for people living with dementia.

### Engagement of people living with dementia in meaningful activities

#### Identifying meaningful activities (past and current)

Participants with dementia widely discussed interests related to domestic activities and daily living: *“I like to work in the backyard, I like to take care of the chickens, I like to cook and do housework”* (Participant with dementia 19). Participants also emphasized intellectual activities like reading, doing crossword puzzles and playing games: *“I have several books from my trips that I've never had the time or inclination to read (…) I used to make my trips when I was younger and now, I'm reading books about the places I've visited”* (Participant with dementia 8). They also mentioned physical activities like walking in the neighborhood: *“During the day, I always find things to entertain myself. I rarely stay at home and it's always on the street that I find things to entertain myself”* (Participant with dementia 21). Some participants showed a preference for creative activities like knitting and photography: *“I like to knit or crochet. It's really my favorite hobby (…) I've been spending more time knitting or crocheting lately. I've been making dolls that I give to friends, especially family children”* (Participant with dementia 16). Religion also seems to play an important role for people living with dementia who participated in the focus groups and interviews: “*I’ve got two extra channels on my TV that I've added, and those are the ones I like (…) it's about religion (…) praying the rosary*” (Participant with dementia 5); “*I'm dedicated, I'm catholic. I'm very dedicated to the church and serving the church”* (Participant with dementia 8).

The activities reported by informal caregivers were similar to those reported by people living with dementia. Informal carers talked about how the people they cared for liked to engage in activities related to what they did throughout their life, and how they were also open to trying new ones: *“Despite everything, she* [Participant with dementia] *still took part in a cut-out activity. She could still see, she had to stop halfway through because her eyes were getting tired, but after a while she started again”* (Informal caregiver 1); “*She* [Participant with dementia] *started seeing some men play dominoes and one day she said to me ‘I’d like to learn’(...) The funny thing about all this is that the grandchildren watch, the sons-in-law watch and they’re all starting to play dominoes as well. She’s very happy because she’s learned to play dominoes”* (Informal caregiver 8).

Similarly, healthcare professionals stressed the importance of identifying meaningful activities related to the individual’s personal life: *“They're looking for what they've always done, right? So, they want to keep going, they want to keep doing what they used to do (…)”* (Healthcare professional 4). The receptiveness to new activities and how they can be meaningful was also discussed: *“The truth is that people also want to try new things. We have several people who have been to the museum for the first time in the context of this program [intervention in a museum context] and what is new is, or can also be, meaningful, pleasurable”* (Healthcare professional 3). One healthcare professional stressed the importance of religion in the population she was working with: *“The question of religiosity. It's still very strong in the group we have (…) and for a wide range of our residents and the people who are also in the daycare unit. It’s something very meaningful”* (Healthcare professional 5).

#### Barriers to engage in meaningful activities

Many participants with dementia talked about giving up activities that were meaningful to them: *“I used to go swimming, now I don’t (...) I used to volunteer, now I don’t either. So, I’m more inactive now”* (Participant with dementia 9). Reasons behind such abandonment were related to both physical factors and changes in cognitive abilities. With respect to physical factors, one participant talked about how she now avoids going for a walk due to her lack of mobility: “*Sometimes they go for walks, they want to take me, and I find it difficult because I have a [medical condition] in my legs. So, I hold back a lot when I go out, because then I have to get into the van”* (Participant with dementia 2). Similarly, one participant spoke about how he stopped doing some of his favorite technology-related activities because he was in a wheelchair: *“I’m in a wheelchair, I have trouble moving around, so (...) I mean, just charging the computer and the cell phone and so on (...) just charging was a hell of a problem! I can’t do it in a wheelchair”* (Participant with dementia 6). Regarding cognition, one participant explained that she kept forgetting what she reads: *“I also remembered my mother saying that she loved to read (…) and I’d say, ‘Mom, are you reading?’, ‘I'm reading, but this is like pouring water into a river’ (…) and now I feel the same”* (Participant with dementia 16). Another participant spoke about how she now had a harder time finding the words in her crossword puzzles which lead to frustration: *“At the moment, I do crossword puzzles, but there is a time I can’t find the words and I give up (…) sometimes I do it, but I don't continue, because I get nervous and put the book down”* (Participant with dementia 19).

Informal carers expressed concern on how the people they cared for seem to have experienced changes in motivation to engage in meaningful activities: “*Now he* [Participant with dementia] *doesn't have the initiative to pick up a book and read or play music or say he wants to play cards or whatever. No, we always have to get him to do something, because if we don’t, when he gets up, he'll sit on the sofa and stay there all day, only getting up to eat*” (Informal caregiver 6). Informal carers also identified individual physical and cognitive barriers for the people they care for: “*She* [Participant with dementia] *had always cooked for herself and her family and from one moment to the next, we noticed that her dementia was progressing. She lost the ability to cook, not so much for sewing, but now towards the end, yes”* (Informal caregiver 4). People living with dementia not engaging in meaningful activities for fear of failing was also discussed: *“She’ll feel bad for making a mistake (…) I don’t know. In my presence, I don’t think she’d feel comfortable doing it [activities]”* (Informal caregiver 2).

Healthcare professionals identified additional environmental barriers, including a lack of appropriate resources: *“It has to do with the lack of activities and resources on offer that are suitable for older people, because, in fact, what often appears [information and activities] is something completely childish”* (Healthcare professional 4). Communicating with people living with dementia was considered very important: *“The person often ends up being excluded before they lose the ability to decide or say anything, and so I think that talking to the person with dementia is absolutely essential”* (Healthcare professional 4). In addition, it was pointed out how adjusting expectations is important when doing activities with people living with dementia: *“It’s just that we can’t expect the activity to last 30 min, it might last 5 (…) so maybe I have to adjust the time clearly, don't I? Anything too long can be painful, can't it?”* (Healthcare professional 8).

### Experiences of people living with dementia with technologies and digital resources

#### Relationship with technology

Most participants with dementia reported using their cell phone to make and receive calls: *“I’m clumsy with a cell phone. I receive and make calls, but apart from that, I don’t have the capacity for anything else”* (Participant with dementia 8). Only three participants reported using this device to access apps: *“I don’t really have the habit of always having my cell phone in my hand, but I do like to check daily news and every now and then I browse Facebook a bit”* (Participant with dementia 16). On the other hand, the computer was used by seven participants for various purposes, including socializing and entertainment: *“I really enjoy going to the computer (…) My daughter is in Australia, and I like to talk to her on the computer, on Skype”* (Participant with dementia 2); *“Those games that are really for the cognitive part, that's mostly what I do on the computer”* (Participant with dementia 17).

Most informal carers reported that the people they cared for were no longer able to use their phones and computers: *“She* [Participant with dementia] *didn’t use it [the Internet] much, but every now and then (…) she would play word games, things like that* (Informal caregiver 1); *“Before, he* [Participant with dementia] *watched videos, he was in a social network group in his area. Now he doesn’t. He just opens the app, picks up the phone sporadically and opens the app and it is whatever pops up in front of him, and then he turns it off”* (Informal caregiver 6).

Healthcare professionals discussed technology use by people living with dementia who attended their institutions and programs, mostly emphasizing the lack of digital skills: *“When thinking about the audience that took part of a fitness program we had, it was mostly older people who didn't have the habit of using a cell phone”* (Healthcare professional 1);*“I don’t have a patient in the cognitive stimulation groups who uses an iPad or computer, no. At most they use a cell phone, but only to make calls to close family members”* (Healthcare professional 7). Age and education level were considered to play a key role in the relationship people living with dementia have with technology: “*Age and level of education. I mean, I think they’re decisive even before we think about whether it’s a website, whether it’s social networks, whether it’s through a tablet, a smartphone or so on*” (Healthcare professional 3). In addition, it was pointed out that most individuals with dementia, who are mostly 75 years and over, still prefer non-digital means of information and communication: *“They still prefer paper and pen”* (Healthcare professional 5). However, it was also stated that a new pattern of more digitally engaged individuals is emerging: *“We're seeing more and more people with dementia at an earlier age (…) and these people have indeed a greater scope for using intervention-based technologies”* (Healthcare professional 2).

#### Accessing information online

Ten participants with dementia used the Internet to see the news or access sources related to their hobbies: *“I joined discussion forums (…) various themes, poetry, photography, the most diverse”* (Participant with dementia 6); *“It’s more on Facebook that I search (...) it’s not techniques, it's more knitting patterns, how to do this or how to do that”* (Participant with dementia 16). One participant used the Internet to look up for information related to his former job: *“From time to time, and this is probably what I do most as a matter of habit, I look at house prices [worked at real estate] and see how the market is evolving”* (Participant with dementia 21).

The Internet can also support carers by allowing them to access information from their own home at any time. Informal carers commented on how they use the Internet to search for activities for the people they care for to keep them active: *“We look for activities that he* [Participant with dementia] *still enjoys, like going to see a soccer match or a concert (…) we search for these activities to get an idea of what’s exists and when”* (Informal caregiver 6); *“I do a lot of research. I look into how to stimulate the brain, how to stimulate the whole component”* (Informal caregiver 8).

Healthcare professionals also discussed Internet use by people living with dementia, emphasizing the search for news and games: *“In addition to social networks, if they already have search habits, for example news (...) it’s down to preferences and what they’re looking for, but I imagine that for some people news is the focus, and games”* (Healthcare professional 5). One healthcare professional pointed out that after the diagnosis people living with dementia tend to prioritize searching information that is related to dementia: *“Issues associated with leisure and meaningful activities come last”* (Healthcare professional 3).

### Preferences on information format

Two participants with dementia expressed clear preferences for the format in which information about meaningful activities should be presented: *“I prefer the text because right now (…) memory is no longer the same. I prefer the text. I can always go back to the text”* (Participant with dementia 16); *“I prefer video (…) it’s more practical and then, when things are important to me, I end up having a notepad next to me to write on, so I don’t forget”* (Participant with dementia 21).

Three informal carers stated they prefer information presented on a video format: *“Okay, I think the video, depending on the activity, can also be more practical for us to understand what the activity is like, how to develop it, how to do it, how to adapt it”* (Informal caregiver 7). One informal carer expressed a strong preference for text: *“It depends on the situation. Q&A can be very useful. Articles too, because they have information, they have more contextualized information, right? Then, of course, maybe even the combination of the two”* (Informal caregiver 1). Although asked to present more details on the reasons why they considered videos to be more practical, they were not willing to elaborate further.

Healthcare professionals pointed out that video can be easier to access and understand and that text should be clear and concise: *“So anything too dense doesn’t work for people with dementia. I’d say it’s the same for caregivers, for one simple reason: time. People don’t have time because they have care demands, because they often have to work and have a million other things to do*”; *“There has to be text, because I understand that you can't get videos for everything. Shorter, simpler sentences. So, there you go, the Q&A format can be a good way, if it’s a really long text it’s going to be confusing, right? So, it must be something more segmented”* (Healthcare professional 8).

## The importance of a support network

People living with dementia reported that they often receive help and support from their family with both technology and activities: *“On the computer, sometimes when I can’t put it on [games], I ask my daughter or son, they put it on and then I play”* (Participant with dementia 21); *“We talked and my granddaughter even gave me a little Christmas game, which is about fitting pieces together so that I can stimulate my head and she sometimes helps me when she comes to my house”* (Participant with dementia 19).

Informal carers spoke about how they tried to motivate the people they care for to keep active and expressed concern related to finding appropriate activities: *“I do my best to encourage her* [Participant with dementia] *to get involved, to do something so she doesn’t lose so much (…) for her to have something to do”* (Informal caregiver 9); *“What activities can I do with him* [Participant with dementia]*? Okay, if he is more limited, how can we adapt some activities to him, right? Considering what he can do on his own or even encouraging him to do so that he maintains some autonomy”* (Informal caregiver 6).

Healthcare professionals discussed the importance of informal carers as mediators: *“Most of the time there is mediation (...) It’s frankly rare, even if the person is at an early stage, for them to show up on their own, because they’ve done their own research [on where to find activities] and came”* (Healthcare professional 3); *“Because families are available and they organize, so families also make the link for technology, right? They’re the ones who often turn on the computer or tablets, who get people to do exercises, and I think that those around our patients are fundamental”* (Healthcare professional 2). They reflected on their own role in facilitating activities and the importance of encouraging people living with dementia to engage in meaningful activities: *“I think it’s up to us, ‘us’, when I say ‘us’ more as technicians, right? As professionals, to encourage the person to continue doing the things they like, maybe we just have to do it in a different way”* (Healthcare professional 8). They also commented on the importance of identifying activities suitable to the individual: *“So, in fact, with regard to what is a meaningful occupation, there is a lot of work done in this area on how to take an occupational history and, in fact, understand what is meaningful for the person (…) it’s always the person who gives it meaning”* (Healthcare professional 4). Other professionals talked about mediating the use of technology: *“In the cognitive stimulation sessions, we use our computers with music, videos and information, but we are the ones using and monitoring the situation”* (Healthcare professional 2); *“We can, in fact, provide a user experience of a platform, of an ‘app’ that is more oriented towards what each person's preferences are and perhaps even help in situations where the person doesn’t have so much use (…) of technology”* (Healthcare professional 5).

## Discussion

This study aimed to understand the needs and preferences of people with early-stage dementia living in Portugal regarding how to receive information on meaningful activities and their relationship with websites and apps pertaining to meaningful activities.

Maintaining engagement in meaningful activities can promote well-being and quality of life for people living with dementia, with activities tailored to an individual presenting a greater impact (Harding et al., 2024). Similarly to Tournier et al. (2023), we found that participants reported a wide range of activities (including technology mediated) which highlights the need to develop a wide and adequate variety of options. Participants with dementia and informal caregivers reported a decrease in engagement in meaningful activities due to reduced mobility (i.e., being in a wheelchair) and cognitive changes (i.e., growing memory complaints). Healthcare professionals identified a lack of appropriate offer on activities and information resources as an important environmental barrier.

In our study, the barriers identified for engaging in meaningful activities are similar to those identified for technology and Internet use. Therefore, digital services developed for people living with dementia should prioritize making meaningful activities suitable for people with reduced mobility and cognitive deficits (Tournier et al., 2023).

In our study, healthcare professionals emphasized age and level of education as decisive factors for Internet use. A literature review on Internet use among older adults (Hunsaker & Hargittai, 2018) linked higher education and income to Internet use. Factors like functional status and cognitive decline were associated with lower Internet use (Hunsaker & Hargittai, 2018), as emphasized by both participants with dementia and informal carers in this study. This outlines the need to further investigate how digital technology may enhance the experience of people living with dementia who might present symptoms that create additional difficulties when using these technologies (Talbot & Briggs, 2022). Technology and internet use among older people in Portugal is progressively increasing; however, in 2019, only 55% of people aged between 55 and 64 were Internet users, with this number decreasing to 34% for people aged 65 and older (Gil et al., 2019). This might explain why we found a low level of Internet use and internet-based meaningful activities in our sample; it might also explain why there was a low level of digital activities identified by participants as meaningful. Understanding how older adults with dementia use the Internet can help clarify how to best support digital media use (i.e., websites, apps) within this population as a clear disparity exists among older adults when making age group comparisons. More specifically, younger old adults are more likely to use the Internet and have better Internet skills (Hunsaker & Hargittai, 2018).

Szabo et al. (2019) found that older adults used the Internet for socializing, which indirectly affected well-being by decreasing loneliness and social engagement. Additionally, older adults used the Internet for instrumental (e.g., banking) and informational purposes, which indirectly improved well-being by increasing engagement in a broader number of activities. This suggests that Internet use can promote engagement in diverse interests and activities among older adults with dementia.

A study that provides an overview of the information needs specifically of people living with dementia and their informal caregivers (Soong et al., 2020) found them to be related to condition-specific information, healthcare service-related information, patient care provision, and caregiver self-care. The interviews/focus groups guide’s questions being more focused on searching for information about meaningful activities might explain why the participants did not address these information needs.

Participants living with dementia talked about their need for technological support from their formal and informal carers. Older adults who receive support from friends and family are more likely to become Internet users, with intergenerational support (i.e., from grandchildren) playing an important role in the adoption of digital technology (Hunsaker et al., 2019). But the approaches to how support is given can result in frustration (Hunsaker et al., 2019). For example, explaining things “too fast” or having a mismatch between what the carers think people living with dementia need and their personal perception of what they need can result in people living with dementia not asking for assistance on future occasions (Peek et al., 2016). In our study, participants with dementia also talked about the role of informal caregivers and healthcare professionals in promoting and facilitating engagement in meaningful activities regardless of its association with digital media or Internet use. This supports the need to include these two groups when further investigating engagement and active participation in meaningful activities for people living with dementia.

Regarding the inclusion of healthcare professionals, the responses came mainly from the psychosocial intervention sector, most likely because they are particularly sensitive to this issue. A Portuguese study that describes the experiences of primary care teams (i.e., general practitioners, practice nurses, social workers) regarding barriers to provide care to people living with dementia, identified that most professionals have a limited view of quality of life and psychosocial needs in this population (Balsinha et al., 2022). These may be a result from a lack of knowledge due to inadequate dementia training, biased observation and prioritizing safety over autonomy.

### Limitations

This study’s prerequisite was recruiting people living with dementia who could use the Internet independently or with support/supervision. Although this was explained to the professionals who helped in the recruitment process, during the first focus groups sessions we realized some participants did not meet this criterion. This might have resulted from the criterion being too ambiguous as we didn’t specify what constituted “using the Internet with support”. Difficulties in the recruitment phase also resulted from the fact that older adults who use the Internet still represent a small proportion of the total older Portuguese population. However, we believe this population will increase soon and become easier to recruit as people living with dementia at an early stage of the diagnosis from coming generations will be more familiar with digital media and the Internet. Additionally, recruiting via institutions may have biased the sample towards older people living with dementia who tend to be less familiar with technology. In future studies, a more diverse sample should be recruited, including young home-dwelling people living with dementia. Additionally, most of our participants had an unspecified type of dementia. In a study that updated the estimated number of community dwellers with dementia in Portugal (Gonçalves-Pereira et al., 2021), 41.9% of cases were diagnosed with Alzheimer’s disease and 30.2% could not be allocated to pure subtypes. It would have been interesting to have clear diagnoses to understand how different types of dementia might impact the ability to engage in meaningful activities and with technology. Finally, several interviews were carried due to the participants’ strong schedule restrictions to be included in a focus group. It is important to acknowledge that using this method might have had an impact on the data collected and consequently on the themes deriving from data analysis. Group dynamics could have generated ideas and responses that might not have been thought of by participants during the individual interviews.

## Conclusion

Most people living with dementia who participated in this study were not familiarized with digital media and Internet use; however, they used computers for socializing and searching for news or information related to leisure activities. A wide range of meaningful activities, including some mediated by technology were identified. Physical and cognitive factors were both associated with low engagement in meaningful activities and technology use. This emphasizes the need to develop digital resources that provide a wide and adequate variety of options of meaningful activities, considering mobility and cognitive challenges. As people living with dementia often rely upon their support network to help with digital media use and to facilitate the engagement in meaningful activities, involving these carers in the development of digital resources is crucial. Digital services that can be used by people living with dementia individually and/or with informal carers and healthcare professionals might ensure broader adoption. Future efforts should prioritize the design of user-friendly interfaces that accommodate the unique needs of people living with dementia, fostering greater autonomy in their digital interactions. By ensuring that digital resources are accessible and enjoyable, these technologies have the potential to improve the quality of life for people living with dementia, allowing them to engage more fully in activities that are personally meaningful and maintain social ties, thus contributing to overall well-being and easing the care demands of caregivers.

## Supplemental Material

Supplemental Material - Exploring the use of digital media to support meaningful activities for people living with dementia: A qualitative study Supplemental Material for Exploring the use of digital media to support meaningful activities for people living with dementia: A qualitative study by Mariana Ramalhete, Rita Maldonado Branco, Soraia Teles, Oksana Tymoshchuk, Rita Oliveira, Joana Quental and Oscar Ribeiro in Dementia

Supplemental Material - Exploring the use of digital media to support meaningful activities for people living with dementia: A qualitative study Supplemental Material for Exploring the use of digital media to support meaningful activities for people living with dementia: A qualitative study by Mariana Ramalhete, Rita Maldonado Branco, Soraia Teles, Oksana Tymoshchuk, Rita Oliveira, Joana Quental and Oscar Ribeiro in Dementia

## Data Availability

In the ethics application, the authors stated that the data would not be shared with anybody outside the research team. Therefore, data will not be uploaded to a repository unless an ethics amendment application is submitted.
