# Supplementary material for: Exploring the use of digital media to support meaningful activities for people living with dementia: A qualitative study
Source: Dementia (London). 2025 Mar 29;25(3):465–81. doi: 10.1177/14713012251330689 (PMC13002935; doi:10.1177/14713012251330689)
Supplement: Supplemental Material - Exploring the use of digital media to support meaningful activities for people living with dementia: A qualitative study [file sj-pdf-1-dem-10.1177_14713012251330689.pdf]

Supplemental File 1 - Semi structured interviews and focus group questions

People with Dementia

1. What are the principal activities (up to three) you like doing daily?
2. Do you look for information on the Internet about activities you like to do? If so, which ones?
3. Where do you look for information on the Internet? Why? What type of information are you looking for?
4. Do you have any difficulty finding information?
5. What could a website/application have to help you carry out those activities?

Informal Caregivers

1. Do the people you care for look for information about activities they like to do? If so, which ones? Do they find it difficult when looking for information on the Internet?
2. What about you? What devices do you prefer using? Why?
3. How do you prefer information to be presented? Why?
4. Do you look for information about activities for the person you care for? If yes, where do you prefer to look for this information?
5. Do you experience any difficulties when searching?
6. Are there any activities that the person you care for enjoy doing that is now more difficult to do? Or any activity that they are afraid of not being able to do in the future?
7. As caregivers, what strategies do you use to help the people you care for to (re)adapt to these activities? Would you like to receive information and/or help with this?
8. As caregivers, what could a website/application have to help you carry out/adapt activities for the person you care for?

Healthcare professionals

1. Which digital information resources do people with dementia prefer to use? Why? What information are they looking for?
2. What are the barriers/challenges when using these digital information resources? What do you consider is missing?
3. What about informal caregivers? Based on your experience with them, which digital information resources do they prefer to use? Why? What information are they looking for? What are the barriers/challenges they face when using digital information resources? What do you consider is missing?
4. After diagnosis, what activities do people with dementia abandon more frequently and/or fear they won't be able to?
5. What strategies do they use to adapt to this change in activities? Do they seek help?
6. What information should we communicate about the activities you have identified as most frequent? How could adaptation strategies be communicated through this platform?
7. On which devices should the platform be available? Why?
8. How should the information be presented to people with dementia? And to informal caregivers? Why?
9. In addition to information on meaningful activities, what other functionalities would the platform have for people with dementia? And for informal caregivers? Why?
