# Supplementary material for: Exploring the use of digital media to support meaningful activities for people living with dementia: A qualitative study
Source: Dementia (London). 2025 Mar 29;25(3):465–81. doi: 10.1177/14713012251330689 (PMC13002935; doi:10.1177/14713012251330689)
Supplement: Supplemental Material - Exploring the use of digital media to support meaningful activities for people living with dementia: A qualitative study [file sj-pdf-2-dem-10.1177_14713012251330689.pdf]

Consolidated criteria for reporting qualitative studies (COREQ): 32-item checklist

Developed from:

Tong A, Sainsbury P, Craig J. Consolidated criteria for reporting qualitative research (COREQ): a 32-item checklist for interviews and focus groups. International Journal for Quality in Health Care. 2007. Volume 19, Number 6: pp. 349 – 357

**No. Item**	**Guide questions/description**	**Reported on Page #**
**Domain 1: Research team and reflexivity**		
Personal Characteristics		
1. Interviewer/facilitator	Which author/s conducted the interview or focus group?	6
2. Credentials	What were the researcher's credentials? E.g. PhD, MD	6
3. Occupation	What was their occupation at the time of the study?	6
4. Gender	Was the researcher male or female?	6
5. Experience and training	What experience or training did the researcher have?	-
Relationship with participants		
6. Relationship established	Was a relationship established prior to study commencement?	-
7. Participant knowledge of the interviewer	What did the participants know about the researcher? e.g. personal goals, reasons for doing the research	6
8. Interviewer characteristics	What characteristics were reported about the interviewer/facilitator? e.g. Bias, assumptions, reasons and interests in the research topic	-
**Domain 2: study design**		
Theoretical framework		
9. Methodological orientation and Theory	What methodological orientation was stated to underpin the study? e.g. grounded theory, discourse analysis, ethnography, phenomenology,	7

	content analysis	
Participant selection		
10. Sampling	How were participants selected? e.g. purposive, convenience, consecutive, snowball	5
11. Method of approach	How were participants approached? e.g. face-to-face, telephone, mail, email	5
12. Sample size	How many participants were in the study?	5
13. Non-participation	How many people refused to participate or dropped out? Reasons?	-
Setting		
14. Setting of data collection	Where was the data collected? e.g. home, clinic, workplace	5,6
15. Presence of non-participants	Was anyone else present besides the participants and researchers?	-
16. Description of sample	What are the important characteristics of the sample? e.g. demographic data, date	Table 1
Data collection		
17. Interview guide	Were questions, prompts, guides provided by the authors? Was it pilot tested?	6
18. Repeat interviews	Were repeat interviews carried out? If yes, how many?	-
19. Audio/visual recording	Did the research use audio or visual recording to collect the data?	6
20. Field notes	Were field notes made during and/or after the interview or focus group?	6
21. Duration	What was the duration of the interviews or focus group?	6
22. Data saturation	Was data saturation discussed?	-
23. Transcripts returned	Were transcripts returned to participants for comment and/or correction?	-
**Domain 3: analysis and findings**		
Data analysis		
24. Number of data coders	How many data coders coded the data?	7
25. Description of the coding tree	Did authors provide a description of the coding tree?	7,8
26. Derivation of	Were themes identified in advance or derived from	7

themes	the data?	
27. Software	What software, if applicable, was used to manage the data?	7
28. Participant checking	Did participants provide feedback on the findings?	-
Reporting		
29. Quotations presented	Were participant quotations presented to illustrate the themes/findings? Was each quotation identified? e.g. participant number	7
30. Data and findings consistent	Was there consistency between the data presented and the findings?	15-17
31. Clarity of major themes	Were major themes clearly presented in the findings?	7,8
32. Clarity of minor themes	Is there a description of diverse cases or discussion of minor themes?	8-15
